# Response of NIH 3T3 Fibroblast Cells on Laser-Induced Periodic Surface Structures on a 15×(Ti/Zr)/Si Multilayer System

**DOI:** 10.3390/nano10122531

**Published:** 2020-12-16

**Authors:** Suzana Petrović, Davor Peruško, Alexandros Mimidis, Paraskeva Kavatzikidou, Janez Kovač, Anthi Ranella, Mirjana Novaković, Maja Popović, Emmanuel Stratakis

**Affiliations:** 1 Department of Atomic Physics, “VINČA” Institute of Nuclear Sciences—National Institute of the Republic of Serbia, University of Belgrade, P.O. Box 522, 11001 Belgrade, Serbia; dperusko@vin.bg.ac.rs (D.P.); mnovakov@vin.bg.ac.rs (M.N.); majap@vin.bg.ac.rs (M.P.); 2 Institute of Electronic Structure and Laser (IESL), Foundation for Research and Technology (FORTH), N. Plastira 100, Vassilika Vouton, 70013 Heraklion, Greece; amimidis@iesl.forth.gr (A.M.); ekavatzi@iesl.forth.gr (P.K.); ranthi@iesl.forth.gr (A.R.); stratak@iesl.forth.gr (E.S.); 3 Jožef Stefan Institute, Jamova 39, 1000 Ljubljana, Slovenia; janez.kovac@ijs.si

**Keywords:** multilayer thin film, ultrafast laser processing, laser-induced periodic surface structure, cell response

## Abstract

Ultrafast laser processing with the formation of periodic surface nanostructures on the 15×(Ti/Zr)/Si multilayers is studied in order to the improve cell response. A novel nanocomposite structure in the form of 15×(Ti/Zr)/Si multilayer thin films, with satisfying mechanical properties and moderate biocompatibility, was deposited by ion sputtering on an Si substrate. The multilayer 15×(Ti/Zr)/Si thin films were modified by femtosecond laser pulses in air to induce the following modifications: (i) mixing of components inside of the multilayer structures, (ii) the formation of an ultrathin oxide layer at the surfaces, and (iii) surface nano-texturing with the creation of laser-induced periodic surface structure (LIPSS). The focus of this study was an examination of the novel Ti/Zr multilayer thin films in order to create a surface texture with suitable composition and structure for cell integration. Using the SEM and confocal microscopies of the laser-modified Ti/Zr surfaces with seeded cell culture (NIH 3T3 fibroblasts), it was found that cell adhesion and growth depend on the surface composition and morphological patterns. These results indicated a good proliferation of cells after two and four days with some tendency of the cell orientation along the LIPSSs.

## 1. Introduction

The role of biomaterials as implants based on natural or synthetic materials in clinical treatment is the replacement of damaged, non-functional organs, and tissues. The selection and manufacturing of biomaterials are predominantly based on imitating the chemical and physical properties of the natural tissue, causing a minimal response of foreign body [1,2]. For years, metallic biomaterials have been widely used in biomedical devices due to an excellent combination of mechanical properties and durability. However, the metallic biomaterials still lack satisfactory biofunctionalities for certain applications, such as blood compatibility for blood-contacting devices and bone conductivity in orthopedic applications [3]. Titanium-based materials are nowadays well integrated into the body, attributing to their constructive properties, such as high specific strength, relatively low Young’s modulus values, excellent corrosion resistance, and good biocompatibility [4,5]. Pure titanium and titanium alloys are ideal as biomaterials due to the superior biocompatibility based on an outer oxide layer with a negative charge at physiological pH and protects dissolving metallic components into biological fluids [6,7]. New biomedical Ti-based alloys have been developed with a high concentration of β-stabilizer elements (β phase of titanium) as potential solutions to the mismatch between the Young’s modulus of the implant and the surrounding hard tissues. The most common alloying elements added to these new alloys are niobium, tantalum, zirconium, and molybdenum, as they do not exhibit any cytotoxic reaction in contact with cells [8,9,10]. 

The surface properties of the biomaterial play critical roles in the interactions between the biological environment and the implant. Surface functionalization is one of particular interest and a requirement to improve the surface bioactivity and other biofunctionalities, in terms of the adsorption of proteins on the material surface, which is determinant for the subsequent processes of cell growth, differentiation, and extracellular matrix formation [11]. Due to the limitation of biocompatibility on the surface properties, thin films and coatings have been considered as a useful alternative. The surface properties of thin films including dissolution and corrosion, fouling resistance, and hydrophilicity/hydrophobicity are modulated to improve the materials response in biological environments [12,13]. The appropriate way for the preparation of Ti-based alloy coatings is the deposition of a multilayer structure with an alternate distribution of Ti and other (Zr, Ta, Mo, and Nb) components in thin films [14,15,16]. These multilayer systems exhibit desirable mechanical properties, because the alloying Ti with mentioned non-cytotoxic metals can be achieved with the further lowering of Young’s modulus [17,18,19,20]. 

The surface modification of the biomaterial aims to create specific chemical and physical properties that offer a favorable cellular response. In cases where tissue integration is desired, the physical environment should include macro, micro, and even nanoscale features that allow for cells to adhere, proliferate, and migrate [21,22,23]. Femtosecond laser texturing holds promise for the surface modification of biomaterials due to a wide application to all materials; the possibility of getting a wide variety of structures with micro- and nano-scaled features; and a fast, repeatable, and contactless process. The femtosecond laser pulses exhibit some advantages such as less debris contamination, reproducibility, precision, and a minimal heat-affected zone, making it a very promising technique for the surface modification of biomaterials [24]. With laser fluence close to the damage threshold for given materials, the nanoscale periodic ripple structures usually are formed due to interference between the incident laser beam and the surface scattered wave [25,26,27,28,29]. The interaction with intense laser pulses involves a lot of physical processes simultaneously (heating, melting, ejection of species, vaporization, shock wave propagation, and expansion) inducing the formation of rougher surface texture-like grooves and spikes. Laser surface modification is a unique method that allows the production of a bioactive surface with formation of the desired oxide and alloy, the creation of nano/micro textures, and a change in the wettability of the surface. The functionalization of these bioactive surfaces is reflected in the adhesion, spreading, and proliferation of the different cells and wherein to inhibit the development of bacterial infections. Surfaces with micro/nano morphological characteristics support cell growth. Furthermore, bacteria adhere preferentially to polished surfaces and to surfaces with cavities larger than the bacteria size, but the development of bacterial infections on the nano-textured surfaces is reduced [30,31,32,33,34].

The orientation of cells is a crucial issue for nerve regeneration and muscle reparation. The cell growth is determined by the structure of the extracellular matrix, where the tri-dimensional patterns can induce the spread of cells in the desired direction. At the surface with roughness determined by randomly distributed features, cells are oriented in all directions. When muscle tissue made up from elongated cells (fibers) are cultured on well-defined patterns with laser-etched micro and nanometer lines, the cells will tend to localize in the grooves between the lines, favoring oriented growth. In the healthy tendons, fibroblasts are oriented in the direction of stretching; however, the fibroblasts tend to orient themselves randomly during the tendon healing. By incorporating an extracellular matrix with suitable patterns, the direction of fibroblast growth and better tendon healing can be influenced [35,36].

Surface modification in terms of the oxidation and texturing of Ti-based materials shows cell repellence explained by limitation of the cell flexibility as well as a limited contact area, which considerably weakens the cell adhesion. On the other hand, morphological features (laser-induced periodic surface structure (LIPSS), spike etc.) reduce adhesion of the trans-membrane proteins in the cell membrane, which are in contact with their ridges, leading also to weaker adsorption. By studying the cell response, it was found that most cells are attached to the tops of morphological objects and do not reach the valleys. In addition, it has been observed that the certain number of cells is oriented to follow the direction of the LIPSS [37,38]. In the future, special attention should be focused on researching the possibility of deposition of Ti-based multilayer thin films and their laser processing, which would achieve an appropriate distribution of composition (alloy and oxide) in a small thickness and the creation of bioactive surface structures.

In this study, the main focus was directed to the examination of a novel titanium–zirconium (Ti/Zr) bimetallic nanocomposite in order to create a bioactive surface with adjusted composition and morphology for cell responses with a high degree of proliferation, as well as cell growth along laser-induced morphological features. Following the previous sub-ablative femtosecond laser studies for the fabrication of a micro/nano laser-induced periodic surface structure (LIPSS) on the multilayer thin films, the formation of an LIPSS was explored for the possible creation of alloys in the multilayer Ti/Zr system and a very thin oxide layer on its surface. The micro/nano patterns distribution with changed composition and wettability are evaluated with the aim of determining their influence in cell proliferation and cell morphology with the possibility of cell orientation along the laser-induced periodic structure.

## 2. Materials and Methods 

### 2.1. Deposition of Ti/Zr Multilayers 

The multilayer structures composed of consecutively distributed titanium and zirconium layers were deposited in a Balzers Sputtron II system, using 1.3 keV argon ions and 99.9% pure Ti and Zr targets. Before deposition, the chamber was evacuated to the base pressure of 1 × 10^−6^ mbar, while the Ar partial pressure during deposition was 1 × 10^−3^ mbar. For the substrate, we selected a silicon wafer Si (100), which was cleaned by etching in hydrofluoric acid (HF) and immersion in deionized water before mounting in the chamber. The deposition of multilayers was performed in a single vacuum run at a deposition rate of 0.17 nm s^−1^ for both Ti and Zr components, without heating of the substrates. The total thickness of the complete multilayer structure, which consisted of fifteen (Ti/Zr) bilayers, was 500 nm, where thickness of individual Ti and Zr layers was about 17 nm.

### 2.2. Laser Processing of Ti/Zr Multilayers

Laser processing of the multilayer 15×(Ti/Zr) thin films was performed with the Yb:KGW laser source Pharos SP from Light Conversion. The surface of thin films was irradiated by focused linearly p-polarized pulses with the following characteristics: repetition rate of 1 kHz, pulse duration equal to 160 fs, central wavelength of 1030 nm, and 43 µm Gaussian spot diameter. Samples were laser processed in an open air ambient environment and mounted on a motorized, computer controlled, X-Y-Z translation stage, at normal incidence to the laser beam. For higher precision, the irradiations were conducted at identical conditions covering a surface of 5 × 5 mm at a pulse energy of 2.5 μJ and scan velocity of 3 mm s^−1^ with constant distance between lines of 15 μm. In each line, the energy per pulse was assumed to be constant, since the pulse energy deviation was less than 1%.

### 2.3. Characterization of Ti/Zr Multilayers

The depth profile in the unmodified and laser-modified areas of the 15×(Ti/Zr)/Si system was analyzed by Auger electron spectroscopy (AES) in a PHI SAM 545 spectrometer. During depth profiling, the sample was sputtered by inclined Ar ion beams of 1 keV at an ion incidence angle of 47° with respect to the surface normal. Surface compositional analysis of the 15×(Ti/Zr)/Si system was done by X-ray photoelectron spectroscopy (XPS) using the PHI-TFA XPS spectrometer. The relative sensitivity factors were used for the calculation of surface concentrations [39], and they were provided by an instrument producer. Detailed surface morphology after irradiation was examined firstly by optical microscopy and then by scanning electron microscopy (JEOL JSM-7500F) equipped with energy-dispersive X-ray spectroscopy (EDS) (Oxford Instruments INCA) and by atomic force microscopy (AFM, Solver PRO 47) in the contact mode with a standard preamplifier. Structural characterization of the samples was done by cross-sectional transmission electron microscopy (TEM) in conventional and high-resolution modes, using an FEI Talos F200X microscope (Thermo Fisher Scientific, 168 Third Avenue, Waltham, MA USA) which was operated at 200 keV voltages. The samples for TEM examination were prepared by a conventional procedure including the cutting and mounting of the samples onto a copper slot, followed by polishing and Ar ions milling. In addition, the samples were also analyzed in scanning transmission (STEM) mode with energy-dispersive spectrometry (EDS) elemental profiling and element color mapping.

### 2.4. Cell Study

Cell integration was studied with the NIH 3T3 established adherent mouse fibroblast cell line (obtained by Mr. G. Vrentzos, IMBB, FORTH). Fibroblast NIH 3T3 cells were grown in cell culture flasks using Dulbecco’s modified Eagle’s medium (DMEM (Invitrogen, Grand Island, NY, USA) supplemented with 10% fetal bovine serum (Biosera, Sussex, UK) in a 5% CO_2_ incubator (Thermo Scientific) at 37 °C. Laser-processed samples were autoclaved and transferred into sterile wells of 24-well plates (Sarstedt; Numbrecht, Germany). Culture medium with 3 × 104 cells were seeded onto the samples, where they were cultured in different time periods, ranging from two to four days in order to estimate the cell orientation, adhesion, and proliferation. The cultured samples with the NIH 3T3 fibroblast were washed twice with 0.1 M sodium cacodylate buffer (SCB) and fixed with 2% glutaraldehyde (GDA) and 2% paraformaldehyde (PFA) in 0.1 M SCB for 30 min. Subsequently, samples were washed twice with 0.1 M SCB and dehydrated in increasing concentrations (from 30 to 100%) of ethanol. Finally, before the determination of NIH 3T3 fibroblasts cell morphology, samples were dried in a critical point drier (Baltec CPD 030), sputter-coated with thin (10 nm) gold/palladium layer (Baltec SCD 050) and observed by Scanning Electron Microscope (JEOL JSM-6390 LV). 

The cytoskeleton, focal adhesion points, and nucleus of NIH 3T3 fibroblasts were stained for actin filaments, vinculin, and 4′,6-Diamidino-2-Phenylindole (DAPI). Specifically, after 2 and 4 days of cell culture, the samples were fixed with 4% paraformaldehyde (PFA) for 15 min and permeabilized with 0.1% Triton ×-100 in phosphate-buffered saline (PBS) for 5 min. The non-specific binding sites were blocked with 2% Bovine Serum Albumen (BSA) in PBS for 30 min. The samples were incubated with α-vinculin primary antibody (FAK 100, Actin Cytoskeleton/Focal Adhesion Staining Kit, Sigma Aldrich) (1:500 in PBS–BSA 1%), and then, the next day, double labeling for 2 h at room temperature with Alexa Flour 48 2nd Antibody, mouse (Thermo Scientific) for focal adhesion points staining, and Phalloidin–Tetramethylrhodamine B isothiocyanate (TRITC-conjugated) phalloidin (FAK 100, Actin Cytoskeleton/Focal Adhesion Staining Kit, Sigma Aldrich) (1:500 in PBS–BSA 1%) for actin filaments in the cytoskeleton staining. Finally, the samples were washed with PBS and put on coverslips with 4′,6-Diamidino-2-Phenylindole (DAPI) (Molecular Probes by Life Technologies, Carlsbad, CA, USA) for nuclei staining. Cell imaging was performed using a Leica SP8 inverted scanning confocal microscope with ×40 objective. Changes in the directional orientation of cell cytoskeleton, “Local gradient orientation” were performed using the Fiji Image-J plug-in “Directionality” [40]. In this way, the data of the structures, presented in the input image (Actin image on the Confocal), in a given direction was extracted and plotted as a polar plot. The amount of cells was normalized with the maximum value and expressed as a normalized amount. For good statistics, ten images of each time point and each area were recorded.

## 3. Results and Discussion

**Figure 1:**
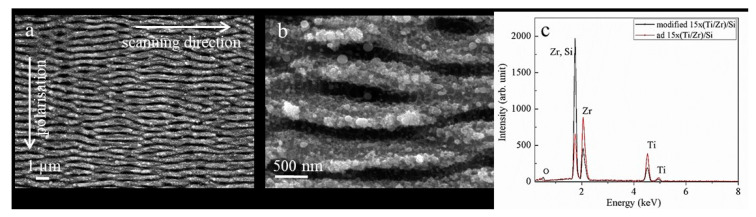
SEM images (**a**,**b**) of laser-modified 15×(Ti/Zr)/Si multilayer thin films with a fluence of 0.662 Jcm*^−^*^2^, and energy-dispersive X-ray spectroscopy (EDS) spectra (**c**) recorded from un-modified and laser-treated surface.

The laser processing of materials with multiple, linearly polarized ultrafast laser pulses is used to create the arrayed surface structure, such as a laser-induced periodic surface structures (LIPSS). An optimal combination of several laser parameters (pulse energy, number of pulses, repetition rate, and scanning rate) has achieved conditions for the generation of LIPSS morphology on the surface of a 15×(Ti/Zr)/Si multilayer system (Figure 1a). The observed surface structure is defined as low spatial frequency LIPSS (LSFL), originating from an interference of the incident laser beam with a surface electromagnetic wave excited during the laser treatment [41]. Polarization of the laser beam was chosen to be normal on the scanning direction, in order to obtain LIPSSs for as long as possible. The relatively good uniformity of the LIPSS can be characterized by their length in the range of 4 to 6 μm, where some LIPSSs are ended, while others appeared or bifurcation points can be recognized. These values of LIPSS length are coincided with the mean free path of excited surface plasmon polaritons (SPP) for Ti component irradiated with laser pulses at a wavelength of 1030 nm [42]. It has been found that titanium has a small SPP mean free path, which supports a good coherence between excited SPP and incident laser radiation, favoring the formation of high regularity LIPSS [42]. The formation of LIPSSs is followed by the ablation of a multilayer Ti/Zr system without visible hydrodynamic features, but ripples are somewhere covered with a nanoparticles dimension up to 100 nm (Figure 1b). The significant ablation of a multilayer Ti/Zr structure, especially Ti as the top layer, is expected due to the applied fluence of 0.662 Jcm*^−^*^2^ being higher than the ablation threshold of 0.228 Jcm*^−^*^2^ for the Ti component [42]. By comparing the EDS spectra before and after the laser irradiation of a 15×(Ti/Zr)/Si multilayer system (Figure 1c), it has been found that approximately half of the multilayer thin film was removed. The concentration of both components Ti and Zr were reduced by about 50%, while the concentration of Si was increased as a contribution of the substrate. At the same time, the content of oxygen was increased, which indicates that the surface oxidation of Ti and Zr has occurred during the laser processing.

**Figure 2:**
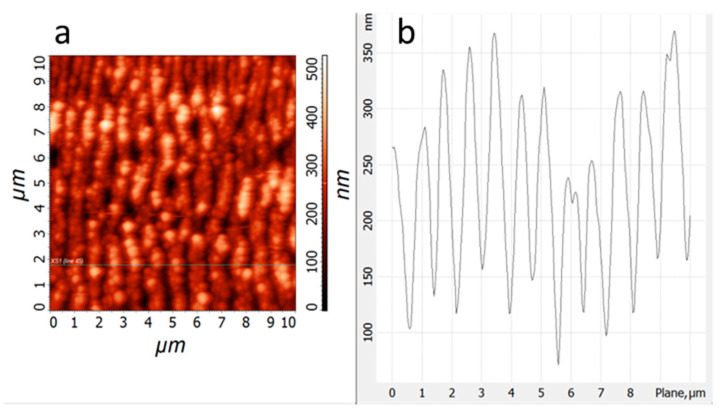
Atomic Force Micrpscopy (AFM) image (**a**) and surface profile (**b**) for laser-treated 15×(Ti/Zr)/Si multilayer thin film at fluence of 0.662 Jcm*^−^*^2^.

The morphological features of the created LIPSS at the surface of the 15×(Ti/Zr)/Si multilayer thin film are specified in more detail by atomic force microscopy (Figure 2). The whole laser-processed surface (5 *×* 5 mm) was covered by uniform distributed LIPSSs (Figure 2a), which are almost regular and identical with lengths in the range from 4 to 7 µm. The consequence of the ripple structure is a higher roughness of the modified surfaces (Ra = 65 nm), causing the higher total surface. The average periodicity between LIPSSs was about 880 nm (Figure 2b), which would correspond to the formation of low spatial frequency LIPSS (LSFL). The depth between ripples reaches a value up to 200 nm (Figure 2b), which would mean that it almost touches the Si substrate at the bottom of the valley between two ripples if half of the multilayer structure was removed.

**Figure 3:**
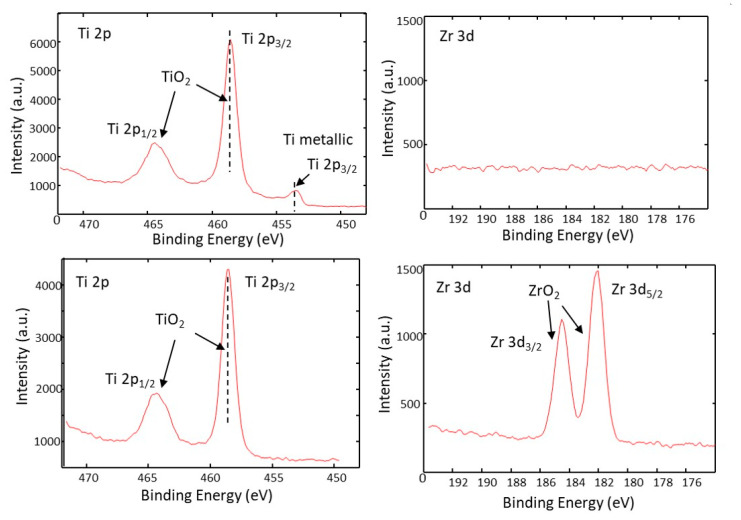
XPS spectra of (**a**) Ti 2p and (**b**) Zr 3d for the surface of as-deposited 15×(Ti/Zr)/Si multilayer thin films and XPS spectra (**c**) Ti 2p and (**d**) Zr 3d recorded after laser modification.

The information about the surface composition and chemical state of constituents (Ti and Zr) before and after laser processing is obtained by an analysis of binding energies in the corresponding XPS spectra. At the surface of the as-deposited 15×(Ti/Zr)/Si multilayer system, where titanium was the top layer (Figure 3a), the signal of the Zr component did not appear (Figure 3b). Titanium on the surface appeared in the oxidation state of +4 with a binding energy of 458.5 eV, corresponding to titanium dioxide (TiO_2_). However, a small amount (approximately 3%) of Ti exists in a metallic state at the binding energy of 454.2 eV. Together with the laser-assisted ablation of the thin film material, laser-induced surface oxidation is also achieved, since both components Ti and Zr exist as oxide phases at a binding energy of 458.5 eV for Ti (Figure 3c) and 182.4 eV for Zr (Figure 3d). The interaction between the laser beam and 15×(Ti/Zr)/Si multilayer thin film generates a dense plasma with lot of energetic species such as Ti^+^, Ti^3+^, and TiO^+^, making the irradiated surface very chemically reactive [43]. The titanium and zirconium react with the gaseous surrounding, forming a surface ultrathin oxide layer rich with carbon due to the decomposition of CO_2_ [44]. On the laser-treated surface, the TiO_2_ constitutes about 58%, while ZrO_2_ covers the surface with 42%, which would mean that slightly more Zr components are removed by laser treatment. The interesting finding was that there was no appearance of silicon at the contact surface, which is very important for cell study: only the biocompatible phases of Ti-oxide and Zr-oxide exist on the surface.

**Figure 4:**
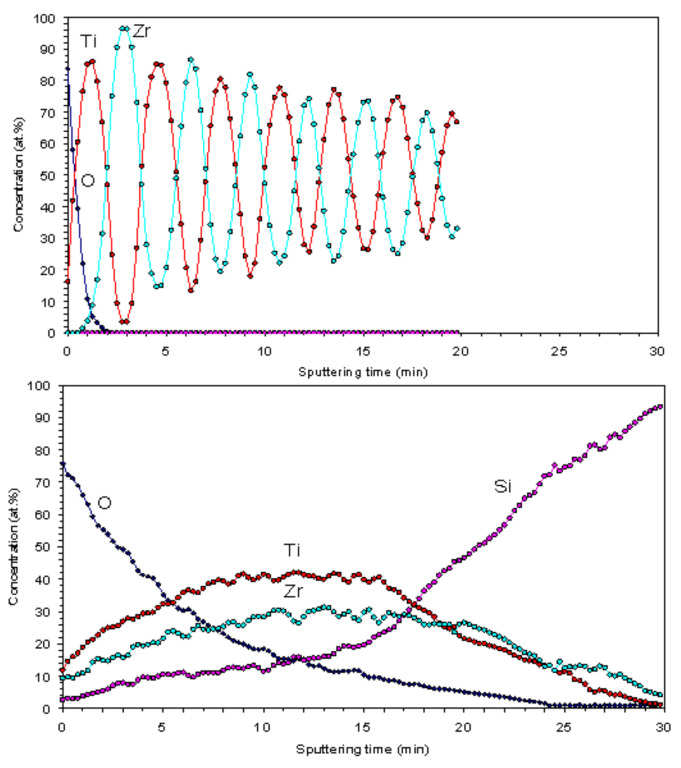
Auger electron spectroscopy (AES) spectra before (**Top**) and after (**Bottom**) laser modification of the 15×(Ti/Zr)/Si multilayer thin film.

The concentration depth profiles through the 15×(Ti/Zr)/Si multilayer system were recorded with AES spectrometry (Figure 4). The spectrum for as-deposited multilayer thin films (Figure 4, Top) is shown to have very well separated Ti and Zr layers with approximately the same thicknesses as the individual layers. Initially well-separated layers are intermixed after the laser irradiation (Figure 4, Bottom), which could be attributed to the alloying between Ti and Zr components. However, a weak periodicity has been retained on curves corresponding to Ti and Zr, indicating that the layers are not completely intermixed. At the surface and near-surface region, the concentration of oxygen was relatively high; this is a consequence of the laser-assisted surface oxidation and the certain penetration of oxygen inside the multilayer structure. On the other hand, silicon atoms were diffused in the multilayer structure and almost reached the surface. 

**Figure 5:**
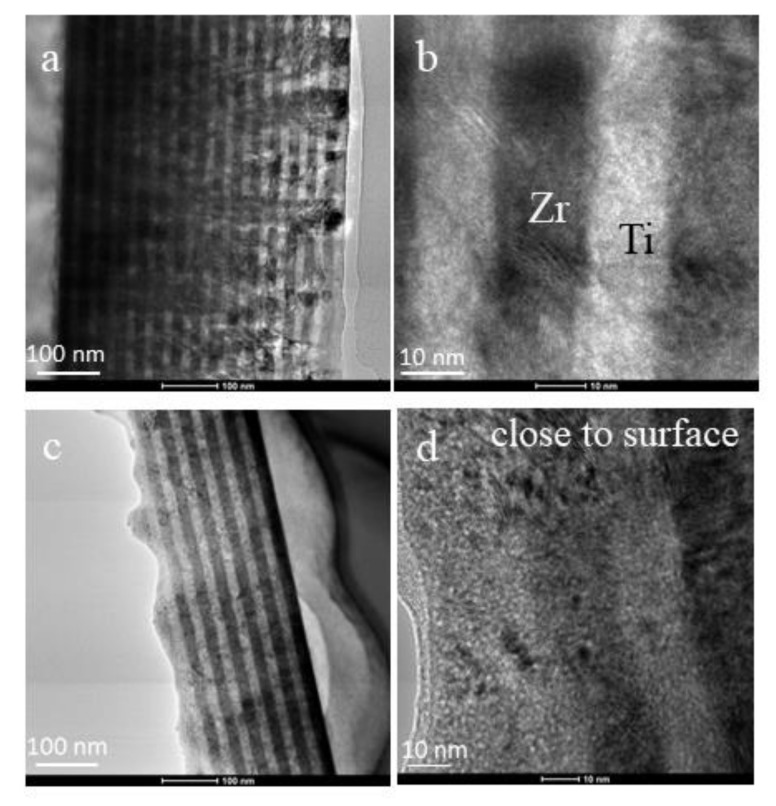
TEM images of a cross-section view for (**a**) a whole unmodified 15×(Ti/Zr)/Si multilayer thin film, (**b**) part of an unmodified structure at high magnification, (**c**) a whole laser-modified 15×(Ti/Zr)/Si multilayer thin film, and (**d**) part of a modified structure close to the surface.

A cross-section view of the 15×(Ti/Zr)/Si multilayer system was obtained with TEM microscopy, which illustrated the volume changes made inside of the thin films after laser processing. A very well-defined multilayer structure composed of a total 30 layers was confirmed for the as-deposited 15×(Ti/Zr)/Si sample (Figure 5a). The thicknesses of individual Ti and Zr layers are identical with values of about 17 nm (Figure 5b). The TEM cross-section view after laser processing (Figure 5c) confirms previous assumptions obtained by the SEM-EDS method that almost half of the multilayer structure was removed, because thirteen layers remained from the deposited thirty. The TEM microphotograph (Figure 5c) has shown that the part of the thin films close to the surface has mixed layers (Figure 5d), but the layers that are close to the Si substrate remain quite clearly separated. A small part of the sub-surface region, including a few layers (no more than three), has very fine grain structures, where Ti and Zr are totally intermixed with a high possibility to form alloy.

**Figure 6:**
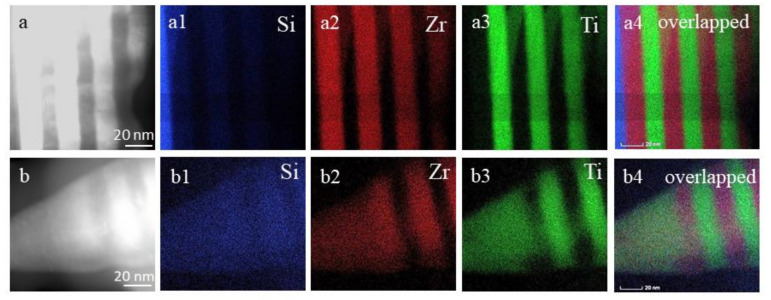
Elemental mapping (Si, Zr, and Ti) and their overlapping for (**a**) an as-deposited, (**a1**) elemental map for Si, (**a2**) elemental map for Zr, (**a3**) elemental map for Ti, (**a4**) overlapped all elemental map and (**b**) laser-modified 15×(Ti/Zr)/Si multilayer thin film, (**b1**) elemental map for Si, (**b2**) elemental map for Zr, (**b3**) elemental map for Ti, (**b4**) overlapped all elemental map

The EDS/TEM elemental mapping images are shown in Figure 6 to identify the spatial distributions of Ti, Zr, and Si components in the multilayer Ti/Zr system before and after laser processing. In elemental maps for the unmodified sample (Figure 6a), it can be found that Si atoms appeared only in a narrow area attributed to the substrate (Figure 6a1), while Zr (Figure 6a2) and Ti (Figure 6a3) atoms were grouped in periodically repeated areas associated with their individual layers. In the case where all three elemental maps were overlapped (Figure 6a4), the edges between the Ti and Zr layers are very sharp, pointing to their complete separation. On the contrary, in the laser-processed sample (Figure 6b), the spatial distribution of Si atoms (Figure 6b1) shows the penetration of Si atoms into the multilayer structure. The spatial distribution of Si atoms reflects that the Si atoms on their tracing through the multilayer structure are more collected in the Zr layer than in the Ti layer. Generally, it is known that Si is the mainly dominant diffusion species, and Si atoms as smaller atoms regarding Ti and Zr are easily diffused in their crystal lattice, locating in interstitially positions [45]. Elemental maps for Zr (Figure 6b2) and Ti (Figure 6b3) reveal that these atoms are intermixed, indicating the final result to the formation of Ti-Zr alloy due to their unlimited dissolving [46,47]. From the image obtained by the mutual overlap of all three elemental maps (Figure 6b4), it can be concluded that the degree of Ti and Zr mixing is reduced with the distance from the surface, whereby they keep a layered structure inside of the thin films.

**Figure 7:**
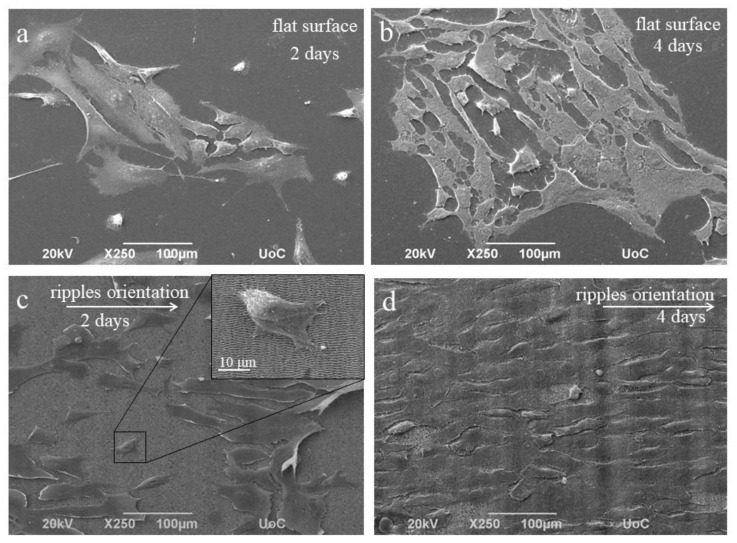
SEM images of NIH 3T3 fibroblasts cultivated on the as-deposited (**a**,**b**), and laser-processed 15×(Ti/Zr)/Si multilayer thin film (**c**,**d**), for 2 and 4 days, respectively.

**Figure 8:**
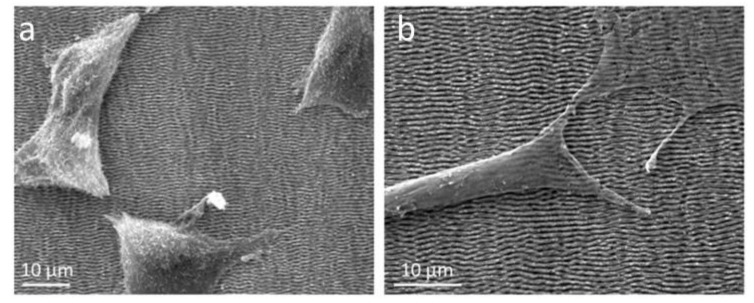
SEM images of NIH 3T3 fibroblasts cultivated on laser-modified 15×(Ti/Zr)/Si multilayer after (**a**) two and (**b**) four days.

The biocompatibility of this laser-patterned thin films is closely related to the cell viability and proliferation with attachment, adhesion, and spreading in the early phase of the cell–material interaction, and at the same time, it demonstrates the role of surface topography in influencing the cell behavior [48]. A morphological examination of NIH 3T3 fibroblast cell proliferation on as-deposited and laser-modified 15×(Ti/Zr)/Si multilayer surfaces was estimated by SEM analysis after two- and four-day cultivation (Figure 7). On the flat area of the as-deposited 15×(Ti/Zr)/Si sample, there is an arbitrary cell growth occurring in all directions (Figure 7a), especially after four days when the number of cells is increased (Figure 7b). After two-day cultivation, the laser-modified surface of 15×(Ti/Zr)/Si with LIPSS structures is partially covered by cells, forming elongated groups (Figure 7c). The fibroblast cells adhered and proliferated on the LIPSSs in 2 days, with tendency for a growth along the LIPSSs orientation. Almost the whole laser-modified surface is covered by cells, with the significant longer groups of cells in the orientation of LIPSSs after 4 days of cultivation (Figure 7d). In addition to the evident directed growth of fibroblast cells on a laser-created surface topography, it can be observed that the surface topography induced faster cell proliferation due to the significantly greater coverage of the surface with higher and longer cell groups. On the other hand, fibroblast cells are oriented along periodic structures, especially after 4 days of cultivation (Figure 8). 

A relatively high degree of cell proliferation is caused by good cell adhesion, which is directly related to the surface characteristics such as composition and morphology. Increasing the surface roughness of the multilayer 15×(Ti/Zr)/Si system, which is reflected in the creation of parallel periodic structures, has a positive effect on cell adhesion. Surface roughness at order of microns increased the effective surface area of cell adhesion. It could be concluded that cells on the laser-modified surface are immobilized, and the cell deposition/adhesion rates increase as a consequence of cells anchored in morphology features especially in sub-micro- and micro-channels [49,50,51].

This study of the laser-modified 15×(Ti/Zr)/Si multilayer demonstrated that in sub-micro features, the ridge width is commonly larger than or equal to the size of a single cell, which is permissive for cell attachment and migration, as well as cell alignment following the geometrical guidance. In contrast, nanometer features are similar to the ECM (extracellular matrix) architectures and typically much smaller than a single cell, consequently inducing cell alignment in a more fundamental way such as mimicking or signaling the cell membrane receptors [52].

**Figure 9:**
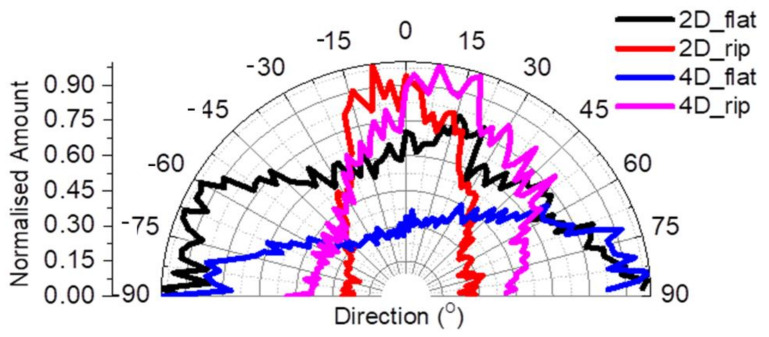
Directional Polar Plot of NIH 3T3 fibroblasts’ cytoskeleton on the flat and ripples area of sample 2 for 2 and 4 days; the black and blue lines represent the flat area at 2 days and 4 days respectively, while the red and magenta lines represent the ripples area at 2 days and 4 days, respectively.

The Directionality Polar Plot demonstrates the effect of the flat and ripples area of the 15×(Ti/Zr)/Si multilayer on the fibroblast cytoskeleton in terms of orientation at both time points (Figure 9). It was observed that there was a random orientation of cells on the flat area at both time points. In particular, the normalized amount of the flat area (black line) after 2 days showed a broad distribution at a range of ±90°, and this was also observed after 4 days (blue line). After 2 days, the fibroblasts oriented on the LIPSS area (red line) with the narrowest distribution ranging from ±15° corresponding to an orientation profile following the LIPSSs direction. The orientation of fibroblasts after 4 days was a little disturbed (pink line), in which the distribution ranged ±30^o^ due to the greater covering of the surface with cells.

## 4. Conclusions

This study included laser processing of the nanocomposite 15×(Ti/Zr)/Si structure in order to achieve surface bioactivation by adjusting the morphology and composition. An optimal combination of laser parameters, a pulse energy of 2.5 μJ and scan velocity of 3 mm s^−1^, achieved conditions for the formation of a high regularity laser-induced periodic surface structure (LIPSS) on a relatively large 5 × 5 mm surface. The formation of an LIPSS was accompanied by an intense ablation of almost half of the Ti/Zr multilayer structure. Laser modification of the nanocomposite 15×(Ti/Zr)/Si samples induced the desired composition changes to achieve optimum biocompatibility. Primarily, the formation of an ultrathin oxide layer on a surface composed of Ti and Zr oxide, and then alloying Ti and Zr components in a sub-surface layer, was reached.

The biocompatibility of the laser-processed nanocomposite 15×(Ti/Zr)/Si thin films is confirmed by a seed NIH 3T3 established adherent mouse fibroblast cell line. The fibroblast cells adhered and proliferated on the laser-induced morphology in 2 days with tendency for a growth along the ripples (LIPSS) orientation. Almost the whole laser-modified surface of 15×(Ti/Zr)/Si system is covered by cells, with the significant longer groups of cells in the orientation of ripples after 4 days of cultivation. The bioactivation of this specific 15×(Ti/Zr)/Si multilayer system with laser surface texturing and an adjusting of surface composition could be potentially useful for tissue engineering and the application of this material as an implant.

The presence of titanium and zirconium oxides at the contact surface with a laser-induced ripple structure enabled physical and molecular interaction between the observed surface and the cell membrane, which contributed to a relatively large number of cells after 2 days and especially 4 days after cultivation. It can be suggested that the newly developed multilayer Ti/Zr thin films achieved a high degree of proliferation and oriented cell growth, and thus, they are suitable implants.
